# Editorial: Metabolic Related Cardiomyopathy in Hyperglycemic Patients

**DOI:** 10.3389/fcvm.2021.826914

**Published:** 2022-01-13

**Authors:** Annalisa Capuano, Emilio Clementi, Giuseppe Paolisso

**Affiliations:** ^1^Department of Experimental Medicine, University of Campania Luigi Vanvitelli, Naples, Italy; ^2^Campania Regional Centre for Pharmacovigilance and Pharmacoepidemiology, Naples, Italy; ^3^Department of Biomedical and Clinical Science “Luigi Sacco” (DIBIC), University of the Study of Milan, Milan, Italy; ^4^Department of Advanced Medical and Surgical Science, University of Campania Luigi Vanvitelli, Naples, Italy; ^5^Mediterranea Cardiocentro, Naples, Italy

**Keywords:** cardiomyopathy, inflammation, heart failure, mitochondrial dysfunction, IL6

An appropriate metabolic milieu plays a key role in the physiological functioning of tissues and cells. This is particularly true for cardiac cells, which depend on extracellular glucose and lipid concentrations to contract and transmit electric signals. An appropriate concentration of both extracellular glucose and lipids is required since extremely low or an exaggerated concentration of these metabolic components might have a deranging effect. In particular, extracellular high glucose concentration has been associated with mitochondrial dysfunction and activation of inflammatory pathways, which are frequently associated with abnormal cardiac structure and function and play a pivotal role in the pathogenesis of heart failure (HF) with reduced and preserved ejection fraction.

## Mitochondrial Dysfunction

The excitation-contraction process consumes a substantial part of ATP produced by mitochondria to support the continuous activity of the heart (1), with a large amount of energy being consumed by the myosin ATPase, SERCAs, and Na/K ATPase (2). Mitochondria cover almost 95% of cardiac cell requests to meet such critical energy requirements through oxidative phosphorylation. Nevertheless, cardiac cells can shift between glucose and lipid oxidation to support a continuous energy output (metabolic flexibility). In normal conditions cardiac cell relies predominantly (almost 60–90%) on fatty acid oxidation to fuel ATP production while ATP derived from pyruvate oxidation covers the remaining part. Upon increased workload, pyruvate is produced in similar amounts by glycolysis and oxidation of lactate. The relative contribution of the glucose and lipid substrate to ATP production can vary greatly depending on energy request, availability, and hormonal/metabolic milieu. *In vitro* in the presence of elevated glucose concentration in the culture medium or *in vivo* in patients affected by type 2 diabetes mellitus morphological and functional mitochondrial changes have been associated with enhanced oxidative stress (3). In particular diabetic cardiomyopathy (DCM) is associated with increased H_2_O_2_ and nitrotyrosine production combined with excessive Ca^2+^ traffic from the sarcoplasmic reticulum (SR)/endoplasmic reticulum (ER) to the mitochondria, which causes mitochondrial calcium overload followed by an electron transport chain uncoupling and excessive free radicals' production (4). The latter effect might be related to an enhanced SE/ER-mitochondria connection through the over-expression of Mfn2 in the absence of changes in other fusion proteins. In addition, in cardiac cells, specific subpopulations of mitochondria can also be more prone to alterations by the occurrence of abnormal metabolic conditions, such as the case of intermyofibrillar mitochondria, typically densely compacted between sarcomeres. The fission-fusion processes can negatively affect their morphology, showing that free radicals imbalance production seems the most damaging factor in DCM.

## Myocardial Inflammation and Diabetic Cardiomyopathy

Myocardial inflammation has been shown to play a critical role in the pathogenesis of HF with reduced and preserved ejection fraction. Systemic and tissue inflammation is very often associated with abnormal cardiac function and structure (5–7). From a physio-pathological point of view, hyperinsulinemia, hyperglycemia, hyperlipidemia, and insulin resistance may conspire to the genesis and worsening of DCM leading to HF. In particular, DCM is associated with cardiac hypertrophy and fibrosis with consequent cardiomyocyte cell death. Those tissue events are also a consequence of a pro-inflammatory cascade (5) encompassing the release of TBF-a, IL1b, and IL-6 and leukocyte activation, associated with an activation of nuclear factor kappa-light-chain enhancer of activated B cells (NF-kB). This may lead to an auto-potentiating vicious cycle that is activated between cardiomyocytes cell metabolic mediated auto-degeneration and pro-inflammatory mechanisms. Among leukocytes, neutrophils secrete various inflammatory mediators such as cytokines and microparticles and neutrophil extracellular traps (NETs), which aggravates the cardiac injury. In addition, macrophages, classified as pro-inflammatory (M1) and anti-inflammatory (M2) cells, lose their balanced ratio with an M1 overactivity. In fact, M1 cells are predominant in type 2 diabetes, worse insulin resistance by secreting resistin and support DCM progression. T-helper cells (either Th-1 or Th-17) also contribute to DCM since they are associated with increased cardiac hypertrophy, fibrosis, and diastolic abnormalities in diabetic children. Finally, NF-kB activation contributes to myocardial fibrosis, hypertrophy, and ventricular dysfunction and is responsible for a reduced RAAS activation. On the other hand, the advanced glycation end product (AGE) and their receptor (RAGE) triggers NF-kB activation, thus aggravating cardiac inflammation.

## Myocardial Dysfunction on Clinical Ground

As whole metabolic and immunological cardiac alterations are responsible for impaired cardiac functioning. Such myocardial dysfunction is associated with DCM and HF with preserved ejection fraction (8, 9). Indeed, the common soil for both latter diseases is an increased cardiac wall rigidity that does not allow the cardiac chamber to receive enough blood by the bloodstream. Over time, cardiac chambers lose their contractility performance, and a HF at reduced ejection fraction develops. Hyperglycemia has also been shown to negatively affect coronary flow reserve, contributing to the decline in cardiac wall performance or leading to ischemic cardiac insult (8–10).

## Author Contributions

AC, EC, and GP contributing to editorial structuring and writing. All authors contributed to the article and approved the submitted version.

## Conflict of Interest

The authors declare that the research was conducted in the absence of any commercial or financial relationships that could be construed as a potential conflict of interest.

## Publisher's Note

All claims expressed in this article are solely those of the authors and do not necessarily represent those of their affiliated organizations, or those of the publisher, the editors and the reviewers. Any product that may be evaluated in this article, or claim that may be made by its manufacturer, is not guaranteed or endorsed by the publisher.
